# Supplementary material for: Modeling glioblastoma heterogeneity as a dynamic network of cell states
Source: Mol Syst Biol. 2021 Sep 16;17(9):e10105. doi: 10.15252/msb.202010105 (PMC8444284; doi:10.15252/msb.202010105)
Supplement: Supplementary file 6 — Source Data for Figure 5 [file MSB-17-e10105-s004.zip › Figure5A_sourcedata/GSEA_3017/hallmarks_stateF.GseaPreranked.1621934628066/HALLMARK_INTERFERON_ALPHA_RESPONSE.html]

Details for gene set HALLMARK\_INTERFERON\_ALPHA\_RESPONSE[GSEA]

|  || Dataset | state33017 |
| Phenotype | NoPhenotypeAvailable |
| Upregulated in class | na\_pos |
| GeneSet | HALLMARK\_INTERFERON\_ALPHA\_RESPONSE |
| Enrichment Score (ES) | 0.4506933 |
| Normalized Enrichment Score (NES) | 1.8356098 |
| Nominal p-value | 0.0072202166 |
| FDR q-value | 0.016523516 |
| FWER p-Value | 0.168 |
Table: GSEA Results Summary

  

Fig 1: Enrichment plot: HALLMARK\_INTERFERON\_ALPHA\_RESPONSE      
 Profile of the Running ES Score & Positions of GeneSet Members on the Rank Ordered List

  

| PROBE | GENE SYMBOL | GENE\_TITLE | RANK IN GENE LIST | RANK METRIC SCORE | RUNNING ES | CORE ENRICHMENT || 1 | IFITM3 |  |  | 38 | 0.987 | 0.0895 | Yes |
| 2 | BST2 |  |  | 55 | 0.910 | 0.1805 | Yes |
| 3 | IFITM2 |  |  | 103 | 0.749 | 0.2402 | Yes |
| 4 | B2M |  |  | 110 | 0.736 | 0.3170 | Yes |
| 5 | PSMB8 |  |  | 230 | 0.568 | 0.3249 | Yes |
| 6 | STAT2 |  |  | 267 | 0.540 | 0.3671 | Yes |
| 7 | TRIM5 |  |  | 305 | 0.508 | 0.4054 | Yes |
| 8 | CSF1 |  |  | 333 | 0.495 | 0.4468 | Yes |
| 9 | LGALS3BP |  |  | 528 | 0.424 | 0.4056 | Yes |
| 10 | PARP14 |  |  | 541 | 0.417 | 0.4452 | Yes |
| 11 | IFIT2 |  |  | 624 | 0.391 | 0.4507 | Yes |
| 12 | SAMD9 |  |  | 876 | 0.330 | 0.3739 | No |
| 13 | IRF9 |  |  | 892 | 0.327 | 0.4025 | No |
| 14 | C1S |  |  | 1087 | 0.294 | 0.3474 | No |
| 15 | TENT5A |  |  | 1117 | 0.289 | 0.3656 | No |
| 16 | TAP1 |  |  | 1317 | 0.266 | 0.3053 | No |
| 17 | PARP9 |  |  | 1344 | 0.262 | 0.3219 | No |
| 18 | PSMB9 |  |  | 1347 | 0.262 | 0.3492 | No |
| 19 | SP110 |  |  | 1384 | 0.258 | 0.3610 | No |
| 20 | UBE2L6 |  |  | 1415 | 0.255 | 0.3751 | No |
Table: GSEA details [plain text format]

  

Fig 2: HALLMARK\_INTERFERON\_ALPHA\_RESPONSE: Random ES distribution      
 Gene set null distribution of ES for **HALLMARK\_INTERFERON\_ALPHA\_RESPONSE**
